# Establishment of a set of St-group wheat-*Thinopyrum ponticum* derivative lines conferring resistance to powdery mildew

**DOI:** 10.3389/fpls.2025.1576050

**Published:** 2025-04-16

**Authors:** Xiaofang Cheng, Yue Guan, Jianing Zhao, Xiaoying Yang, Guangyi Wang, Tingdong Li, Pingchuan Deng, Chunhuan Chen, Jixin Zhao, Changyou Wang, Xinlun Liu, Wanquan Ji

**Affiliations:** ^1^ College of Agronomy, Northwest Agriculture and Forestry University, Yangling, China; ^2^ State Key Laboratory for Crop Stress Resistance and High-Efficiency Production, Northwest Agriculture and Forestry University, Yangling, China

**Keywords:** *Thinopyrum ponticum*, ISH, alien derivatives, wheat powdery mildew, St-group-specific markers

## Abstract

*Thinopyrum ponticum* (2*n* = 10*x* = 70, E^e^E^e^E^b^E^b^E^x^E^x^StStStSt), a wild relative of common wheat (*Triticum aestivum* L., 2*n* = 6*x* = 42), possesses extensive genetic diversity. The primary objective of this study was to develop and evaluate alien derivatives carrying the St-chromosome from *Th. ponticum*, aiming to improve wheat disease resistance and agronomic traits. In this study, a set of St-chromosome alien derivatives was developed from *Th. ponticum*. Chromosomal compositions, karyotypes and homoeologous group affiliations of seven derivatives were characterized using genomic *in situ* hybridization (GISH), fluorescence *in situ* hybridization (FISH), multicolor GISH (mc-GISH), and liquid chip analysis. Resistance assessments showed that the 4St and 7St derivatives exhibited strong resistance to the E09 isolate at the seedling stage and to naturally occurring pathogen mixtures in the field at the heading stage. The 7St derivative line, based on agronomic trait evaluations, is considered an ideal bridging material for breeding, with a reduction in plant height to 71.3 cm, while thousand-kernel weight and kernel length increased to 41 g and 0.77 cm, respectively. Specific markers for the St-homoeologous groups were developed through genome sequencing, achieving a development efficiency of 47.5%. This study provides a theoretical and technical basis for applying *Th. ponticum* genetic resources to improve wheat resistance and agronomic performance.

## 1 Introduction

Bread wheat (*Triticum aestivum* L., 2n = 6x = 42, AABBDD) is one of the world’s top three cereals, providing 20% of global protein and energy. However, wheat production faces numerous biotic and abiotic challenges, including over 200 documented pests and diseases, about 50 of which significantly impact yields and economies (Liu et al., 2022). Modern breeding practices have led to a genetic bottleneck, restricting further improvements (Tanksley and McCouch, 1997; Heerwaarden et al., 2011). Crop wild relatives (CWR) offer a rich gene pool for introducing beneficial allelic variation into wheat.

Powdery mildew, caused by *Blumeria graminis* f. sp. *tritici* (*Bgt*), is a major fungal disease threatening wheat production globally. The pathogen infects leaves, stems and spikes, often leading to the breakdown of resistance genes (Juroszek and von Tiedemann, 2013; Zeng et al., 2014). Known resistance genes in wheat include all-stage resistance and adult-plant resistance. The widely used *Pm8* gene, located on the T1BL.1RS translocation lines, provided adult-plant resistance (Hartl et al., 1992). Varieties carrying the *Pm8* gene, such as Kavkaz and Disponent, have been widely cultivated for large-scale production in China (Zeller and Hsam, 1996; Purnhauser et al., 2011). However, due to the widespread use of this resistance source and the variability in pathogen virulence, the resistance conferred by the *Pm8* gene has been lost (Hurni et al., 2014; Zhao et al., 2016). Currently, most major wheat cultivars in China rely on a few resistance genes, such as *Pm21*, an all-stage resistance gene (Huang et al., 2023). Identifying and utilizing diverse resistance genes is thus essential for developing wheat with durable and broad-spectrum resistance.

*Th. ponticum* (2*n* = 10*x* = 70 (Podp.) Barkworth & D. R. Dewey), an important species in the *Thinopyrum* genus, is composed of the E^e^E^e^E^b^E^b^E^x^E^x^StStStSt. The St genome originates from *Pseudoroegneria* sp*icata*, while the E^e^ and E^b^ genomes derive from *Th. elongatum* and *Th. bessarabicum*, respectively, with significant homology between E^e^ and E^b^ (Zhang et al., 1996). As a robust perennial wild relative, *Th. ponticum* has a rich repertoire of broad-spectrum resistance genes, including those associated with yield-related features (Kuzmanović et al., 2016), abiotic stress tolerance (Wang et al., 2010; Qin and Qin, 2016), as well as resistance to various wheat diseases like leaf rust (Sepsi et al., 2008), stem rust (Friebe et al., 1994; Niu et al., 2014), stripe rust (Wang et al., 2020a, 2022), powdery mildew (Yang et al., 2022, 2023), Fusarium head blight (Oliver et al., 2006; Wang et al., 2020b). Its ability to cross with wheat has made it a key donor for breeding programs for decades (Li et al., 2008).

In 2014, Zheng produced five Xiaoyan-series wheat-*Th. ponticum* partial amphiploids (Xiaoyan 68, Xiaoyan 693, Xiaoyan 784, Xiaoyan 7430, and Xiaoyan 7631). Among them, Xiaoyan 68, Xiaoyan 784, and Xiaoyan 7430 had high resistance to nine stem rust races and have potential as sources of stem rust resistance in wheat breeding (Zheng et al., 2014). In 2022, Jia identified *Th. ponticum* chromosomes introduced into Xiaoyan 81 from Xiaoyan 7430, clarified their homoeologous groups and stripe rust resistance using *in situ* hybridization and the wheat 660K SNP array, and developed chromosome-specific markers (Jia et al., 2022). Zhao demonstrated that Xiaoyan 693 contributed 16.97% of favorable QTL alleles in ‘Kenong 9204’, 171.52% higher than expected (Zhao et al., 2023). Despite such progress, the complex polyploid nature of *Th. ponticum* has hindered genome sequencing, necessitating systematic analyses of its subgenomes and derivatives within wheat backgrounds.

The St-chromosome genome, derived from diploid *P.* sp*icata*, is critical for understanding subgenome composition, tracking agronomically important alien chromosome segments, and advancing gene mapping and recombination studies. While *in situ* hybridization is a widely used technique for detailed chromosome karyotype analysis (Li et al., 2021; Li et al., 2022; Liu et al., 2023a), research on the chromosomes of *Th. ponticum* has advanced via FISH. Li’s investigation of *Th. ponticum* and its progenitor species revealed that the Internal Transcribed Spacer (ITS) regions have experienced both interlocus and intralocus concerted evolution (Li and Zhang, 2002). Kruppa utilized mcGISH to analyze the chromosomal composition of *Th. ponticum* and the synthetic hybrid between *Thinopyrum intermedium* and *Th. ponticum* (Kruppa and Molnár-Láng, 2016). Despite these advancements, the establishment of St-chromosome FISH karyotypes for *Th. ponticum* has not been reported.

Traditional cytogenetic methods are labor-intensive and operationally demanding. Advances in second-generation sequencing technologies, such as specific length amplified fragment sequencing (SLAF-seq), enable high-throughput marker development, offering efficiency and cost-effectiveness for tracking alien DNA (Tong et al., 2022; Liu et al., 2018, 2023b). Liquid chip technology also provides high-resolution tools for genetic analysis (Guo et al., 2023). For instance, Deng’s GenoBaits^®^WheatplusEE efficiently identified chromosome breakpoints in wheat backgrounds (Deng et al., 2024). This study aimed to: (1) clarify the St-chromosome composition of *Th. ponticum* using *in situ* hybridization; (2) determine the homoeology of alien chromosomes with a liquid chip-based GBTS system; (3) evaluate powdery mildew resistance and morphological traits; and (4) develop St-chromosome-specific markers.

## 2 Materials and methods

### 2.1 Plant materials

The plant materials used in this study included *Th. ponticum* (2*n* = 10*x* = 70, PI = 531737), *P.* sp*icata* (2*n* = 2*x* = 14, StSt, PI =236681), *Th. elongatum* (2n = 2x = 14, EE), and the wheat cultivars 7182, Abbondanza (Abb), Chinese Spring (CS) and Shaanyou 225 (SY225). Additionally, seven wheat-*Th. ponticum* substitution/addition lines (CH88, CH94, CH96, CH155, CH157, CH159 and CH161) were included. In 2009, *Th. ponticum* was crossed with 7182 (Abb). The F1 generation was then backcrossed to 7182 (Abb) and subjected to multiple generations of self-pollination. Through several years of phenotypic and cytological analyses, systematic selection led to the development of the seven derived lines. CH88, CH155, CH157, CH159 and CH161 originated from crosses between *Th. ponticum* and 7182, while CH94 and CH96 were derived from crosses between *Th. ponticum* and Abb. All plant materials were preserved in the Wheat Distant Hybridization and Chromosome Engineering Laboratory, College of Agronomy, Northwest A&F University.

### 2.2 Chromosome preparation and *in situ* hybridization

Chromosome spreading from root tips was conducted using a modified dropping method (Han et al., 2004). For each substitution/addition line, at least six plants were analyzed. Sequential genomic *in situ* hybridization (GISH), multicolor fluorescent *in situ* hybridization (mc-GISH), and genomic DNA (gDNA) extraction were performed following the methods of Wang et al (Wang et al., 2020c). *Th. ponticum* and *P.* sp*icata* gDNA were labeled with fluorescein-12-dUTP, while *Th. elongatum* gDNA was labeled with Texas Red-5-dUTP, serving as probes for GISH and mc-GISH. Sheared CS gDNA (heated to 120°C) was used as a blocking agent (Fu et al., 2012). Oligonucleotide probes (Oligo-pTa535-1 and Oligo-pSc119.2-1) were used for FISH analysis (Tang et al., 2014). Chromosomes were counterstained with 4′,6-diamidino-2-phenylindole (DAPI). Hybridization signals were captured with an Olympus BX63 fluorescence microscope equipped with a Photometrics SenSys CCD DP80 camera (Olympus, Tokyo, Japan).

### 2.3 Wheat-*Th. elongatum* liquid array analysis

A wheat-*Th. elongatum* liquid array (GenoBaits^®^WheatplusEE) was used to identify alien chromosomes and fragments from *Thinopyrum* species. gDNA from CH88, CH94, CH96, CH155, CH157, CH159 and CH161 was extracted using the cetyltrimethylammonium bromide (CTAB) method (Porebski et al., 1997). DNA quality and concentration were assessed using the NanoDrop 2000 spectrophotometer (Thermo Scientific, Waltham, MA, USA). DNA library construction, probe hybridization and high-throughput sequencing followed established protocols (Guo et al., 2021). Raw sequencing data were processed using Trimmomatic (v.0.39, Illumina) (Bolger et al., 2014) to obtain high-quality clean reads, which were aligned to the mixed genomes of wheat and *Th. elongatum* using BWA (Li, 2012). Genetic distances were calculated from allele data using GenoBaits^®^WheatplusEE panels, and read depth was normalized with bamdst v.1.0.6 (https://github.com/shiquan/bamdst). Graphs were generated using the R software package.

### 2.4 Powdery mildew infection and evaluation

Seedling resistance was evaluated annually from 2021 to 2024. The assessments were conducted in a controlled environment chamber set at 22°C, with a 16-hour photoperiod and light intensity of 400-1,000 μmol m^-2^ s^-1^. The Bgt isolate E09 was used for inoculation (Zhou et al., 2005). Following the protocol of Lu et al (Lu et al., 2020), seedlings at the three-leaf stage were inoculated, with SY225 serving as the susceptible control. Ten days post-inoculation (dpi), infection types (ITs) were evaluated on a scale of 0-4, classifying plants as resistant (R, IT 0-2) or susceptible (S, IT 3-4).

Heading plant resistance was assessed during the 2021-2024 growing seasons under natural field conditions without artificial inoculation. The experimental materials were planted at Northwest A&F University’s experimental base. Twenty seeds each of 7182, Abb and the seven derived lines were sown alongside SY225. Resistance was scored when SY225 displayed severe disease symptoms, using a scale of 0-9, where 0-4 indicated resistance and 5-9 indicated susceptibility (Wang et al., 2020a).

### 2.5 Evaluation of agronomic traits

Agronomic traits were evaluated during the 2021-2024 growing seasons at Northwest A&F University. Twenty seeds of each material were sown in two rows (1 m long), with 10 cm between seeds and 30 cm between rows. The plant height (cm), awn type, Spikelets per spike, Thousand kernel weight (g), Kernels per spike, Kernel length (cm) and Kernel width (cm) were determined. The following methods were used to measure each trait: plant height was measured from the base of the stem to the top spikelet; awn type includes long awn, short awn, awnless, and top awn; spikelets per spike refers to the total number of spikelets on the main spike; kernels per spike refers to the total number of kernels on the main spike; thousand-kernel weight, kernel length, and kernel width were measured using an SC-G seed testing instrument (Wseen Detection Technology Co., Ltd., Hangzhou, China). Statistical analysis was performed using independent-sample t-tests in SPSS software. Differences between derived lines and the wheat parent were considered significant at p < 0.05, indicated by * (p < 0.05) and ** (p < 0.01).

### 2.6 St-chromosome-specific molecular markers development

The *P.* sp*icata* genome was sequenced using the SLAF-seq technique on the MGISEQ platform (MGI Tech Co., Ltd.) (PE150). SLAFs were filtered using BWA, excluding sequences homologous to CS (IWGSC RefSeq v2.1) and retaining those with >95% similarity to *Th. ponticum* (unpublished genome) as St-specific. Primers were designed using Primer3 Plus (http://www.primer3plus.com/) and synthesized by AuGCT DNA-SYN Biotechnology Co. (Beijing, China). Primers were tested on gDNA from 7182, Abb, *Th. ponticum*, *P.* sp*icata* and the alien lines. PCR reactions (10 µL) included 1 µL template DNA (200 ng/µL), 1 µL of each primer (5 µM), 0.8 µL dNTP mixture (2.5 mM), 1 µL 10× PCR buffer (Mg²^+^ free), 0.12 µL Taq DNA polymerase (500 U, TIANGEN) and 6.08 µL distilled water. The PCR conditions were: pre-denaturation at 94°C for 3 minutes, followed by 35 cycles of 94°C for 30 seconds, 60°C for 45 seconds and 72°C for 25 seconds, with a final extension at 72°C for 10 minutes. PCR products were separated on 1.5% agarose gels in 1×TAE buffer. Specific bands were observed in *Th. ponticum*, *P.* sp*icata* and alien derivatives, but not in 7182 or Abb. Finally, agarose gel images were captured using SensiCapture software (Peiqing Science and Technology Co., Ltd), followed by layout formatting using Adobe Illustrator CC2018.

## 3 Results

### 3.1 Creation and establishment of seven wheat-*Th. ponticum* alien derivatives

**Figure 1:**
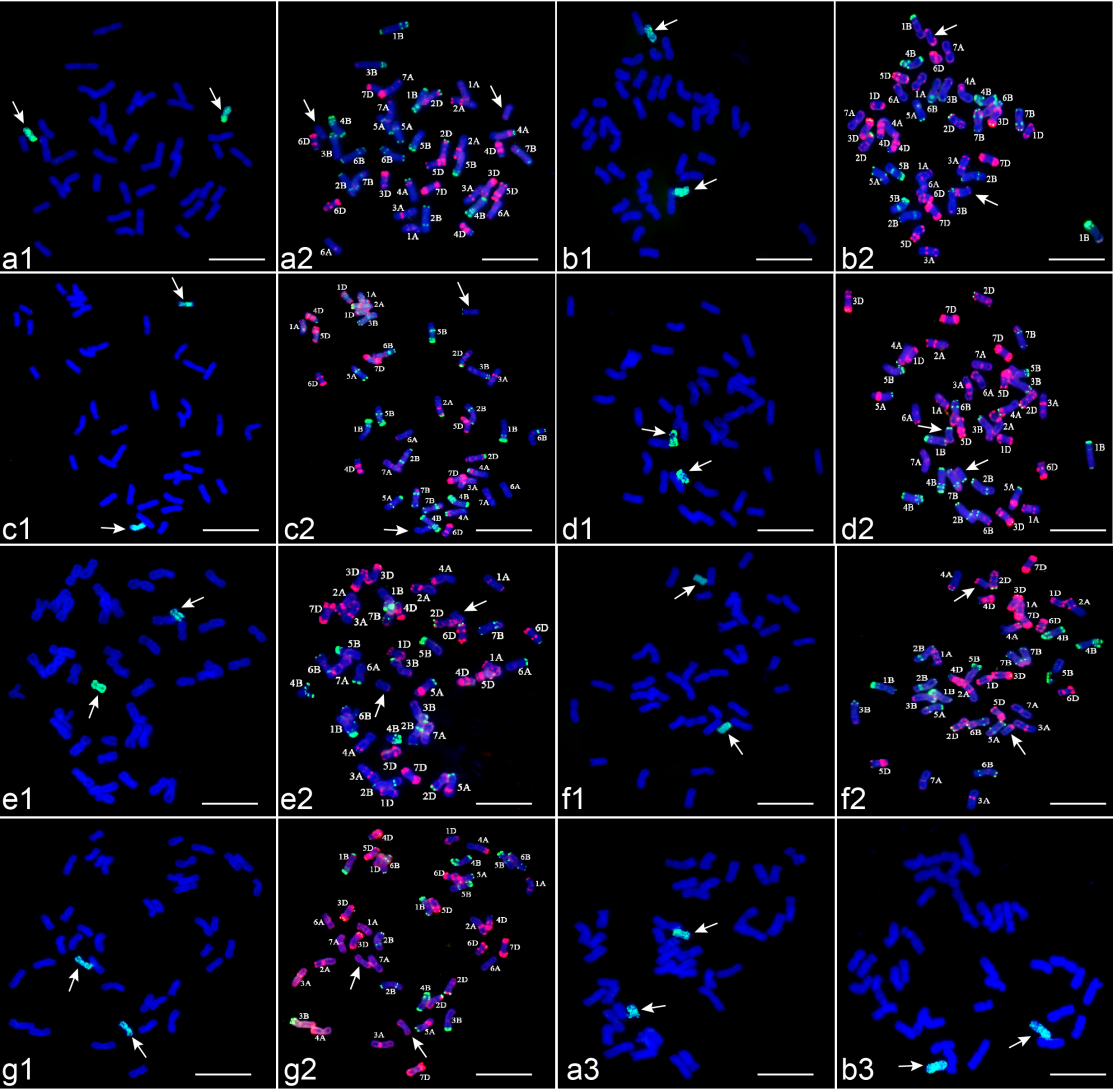
Seven wheat-*Th. ponticum* alien derivatives identified by GISH with *Th. ponticum* gDNA (green) as a probe and FISH with pTa535-1 (red) and pSc119.2-1 (green) as probes and mc-GISH with *P. spicata* gDNA (green) and *Th. elongatum* gDNA (red) as probes. **(a1–g1)** are GISH patterns of derivatives; **(a2–g2)** are FISH patterns of derivatives; **(a3–b3)** are mc-GISH patterns of derivatives. Arrows denote alien chromosomes. **(a)** CH88, **(b)** CH94, **(c)** CH96, **(d)** CH155, **(e)** CH157, **(f)** CH159, **(g)** CH161, Bar = 10 μm.

GISH analysis, using *Th. ponticum* gDNA as the probe and CS gDNA as the block, identified seven wheat-*Th. ponticum* alien lines. Six lines (CH88, CH94, CH96, CH155, CH159 and CH161) exhibited 42 chromosomes, including two from *Th. ponticum* and 40 from wheat (**Figures 1a1-d1, f1-g1**). CH157 contained 21 pairs of wheat chromosomes and two from *Th. ponticum* (**Figure 1e1**). These results classified CH88, CH94, CH96, CH155, CH159 and CH161 as disomic substitution lines, while CH157 was identified as a disomic addition line.

**Figure 2:**
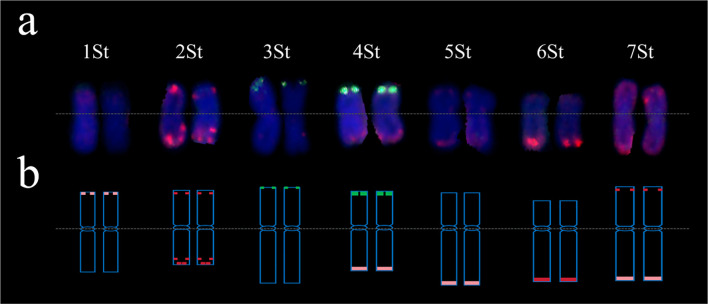
FISH-based karyotype of the *P.* sp*icata* chromosomes using probes oligo probes pTa535-1 (red) and pSc119.2-1 (green). **(a)** FISH-base hybridization patterns of 1St-7St homoeologous groups. **(b)** FISH-base idiogram of 1St-7St homoeologous groups.

Sequential GISH-FISH analysis revealed the substitution or addition of *Th. ponticum* chromosomes in these lines. CH88 exhibited 1D substitution by *Th. ponticum* chromosomes, showing weak pTa535-1 signals at the short arm termini, distinct from other wheat chromosomes (**Figure 1a2**). CH94 contained a disomic substitution 2St (2A) (space), marked by bright pTa535-1 signals at both arm ends and the sub-terminal region of the long arm (**Figure 1b2**). In CH96 (**Figure 1c2**) and CH155 (**Figure 1d2**), 3D and 4D chromosomes were substituted by *Th. ponticum* chromosomes, showing pSc119.2-1 signals at their telomeric regions, with stronger signals in CH155. CH157 retained all 42 wheat chromosomes alongside two *Th. ponticum* chromosomes with faint pTa535-1 signals at the long arm termini (**Figure 1e2**). Reciprocal translocation events were observed at the distal ends of chromosomes 6BL and 6DL, with chromosome 4BS showing breakage and translocation to the chromosome 6AS. In CH159 (**Figure 1f2**) and CH161 (**Figure 1g2**), *Th. ponticum* chromosomes replaced 6A and 7B, respectively, showing pTa535-1 signals on either arm. The St-chromosome FISH karyotype of *Th. ponticum* was confirmed (**Figure 2a**), and a chromosomal idiogram was constructed (**Figure 2b**).

Mc-GISH analysis further confirmed that all seven lines contained two St chromosomes from *Th. ponticum*, with strong green hybridization signals using *P.* sp*icata* gDNA probes (**Figure 1a3-b3**, **Supplementary Figures S1, c3-g3**). The combination of sequential FISH-GISH and mc-GISH characterized the chromosomal composition for all lines.

### 3.2 GenoBaits^®^WheatplusEE array analysis

**Figure 3:**
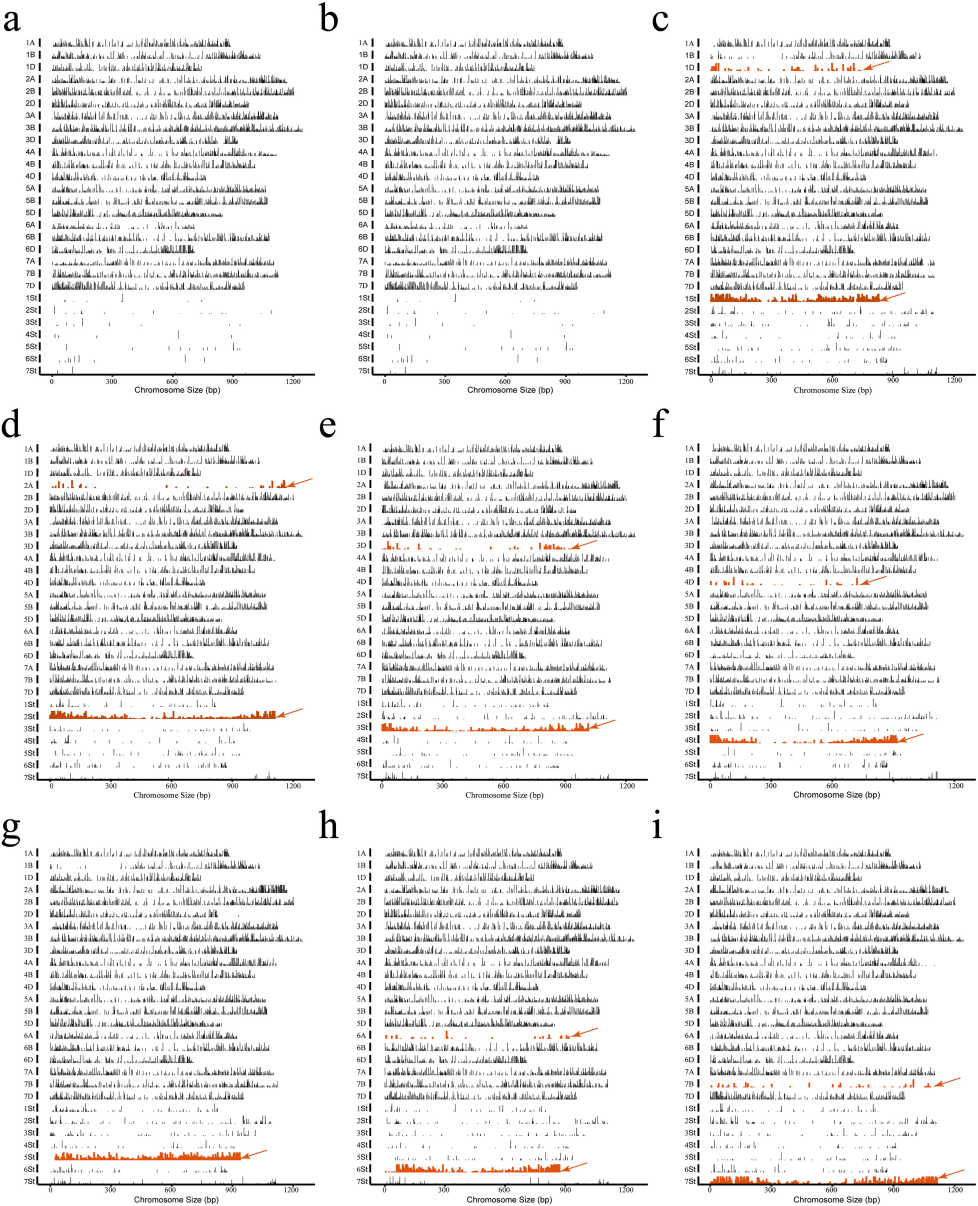
Analysis of the homoeologous groups for exogenous chromosomes in seven wheat-*Th. ponticum* alien derivatives and their common wheat parental lines was performed using the GenoBaits^®^WheatplusEE liquid array. **(a)** 7182, **(b)** Abb, **(c)** CH88, **(d)** CH94, **(e)** CH96, **(f)** CH155, **(g)** CH157, **(h)** CH159, **(i)** CH161. The horizontal axis indicates the physical position in Mb, while the vertical axis indicates the distribution and abundance of sequencing depth per million along the chromosomes. Orange arrows indicate signals of absent wheat chromosomes or enriched alien chromosomes identified using the GenoBaits^®^WheatplusEE.

GenoBaits^®^WheatplusEE array analysis identified the homoeologous groups of alien chromosomes in the derived lines. A total of 45,135 target regions were detected, comprising 5035 loci on 21 wheat chromosomes and 40,100 loci on 7 *Th. elongatum* chromosomes (**Supplementary Table S1**). For 7182 (**Figure 3a**) and Abb (**Figure 3b**), signals were evenly distributed across 21 wheat chromosomes, with low-abundance signals on the 1St-7St chromosomes of *P.* sp*icata*. CH88 exhibited enriched signals along 1St and 20 wheat chromosomes, with sparse signals on 1D and other *P.* sp*icata* chromosomes, confirming the substitution of 1D by 1St (**Figure 3c**). Similarly, CH94, CH96, CH155, CH159 and CH161 were identified as disomic substitution lines for 2St (2A), 3St (3D), 4St (4D), 6St (6A) and 7St (7B), respectively (**Figures 3c–f, h, i**). CH157 was confirmed as a 5St disomic addition line (**Figure 3g**). Signal loss on 1BS in CH88 and CH157 likely resulted from chromosomal structural variations.

### 3.3 Resistance to powdery mildew

**Figure 4:**
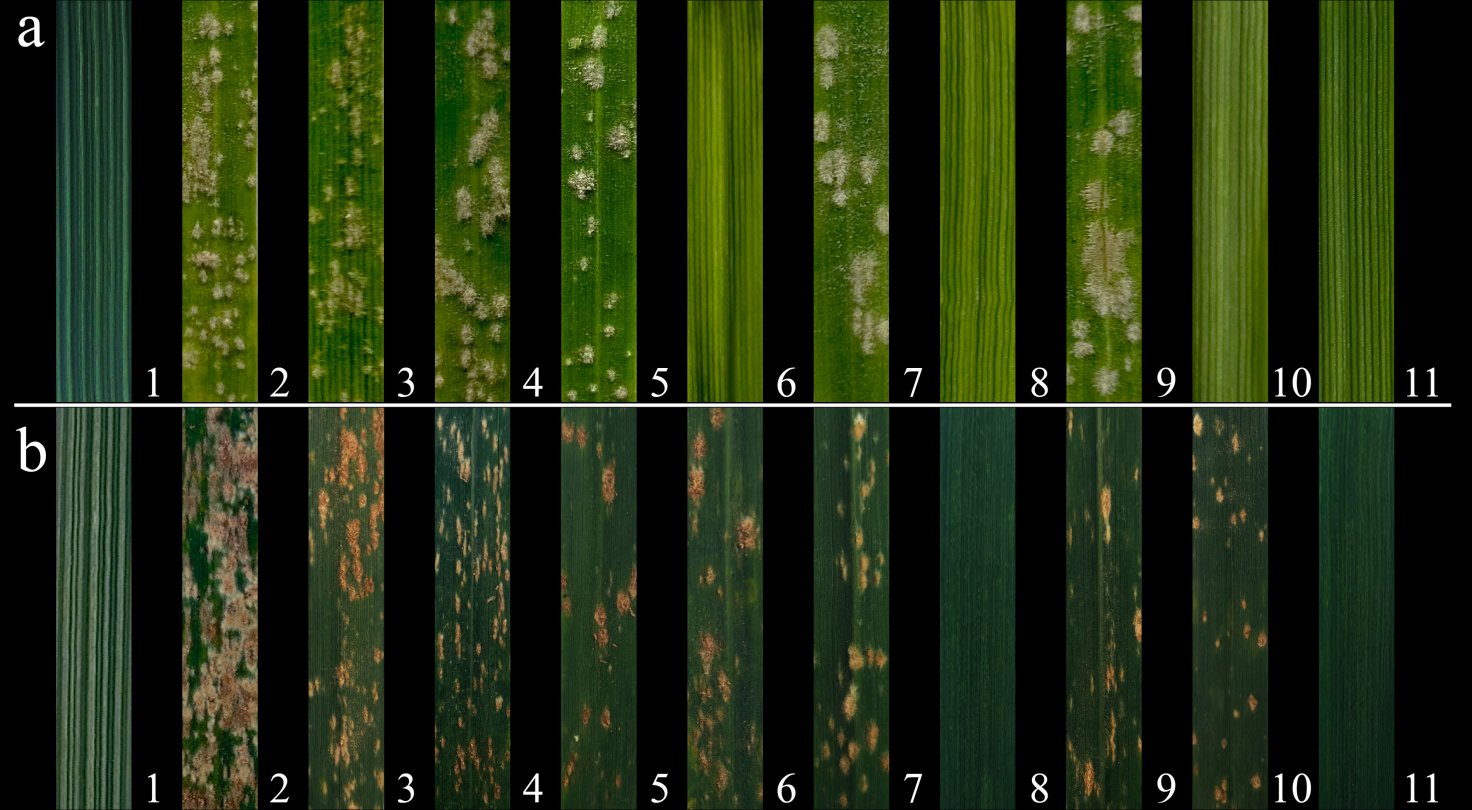
Evaluation of powdery mildew resistance in seven wheat-*Th. ponticum* alien derivatives and their parental lines in 2023-2024. **(a)** Responses to the *Bgt* isolate E09 at the seedling stage. The results of seedling stage resistance evaluation are as follows: 1: *Th. ponticum* (IT = 0/R), 2: SY225 (IT = 4/S), 3: 7182 (IT = 3/S), 4: Abb (IT = 3/S), 5: CH88 (IT = 3/S), 6: CH94 (IT = 0;/R), 7: CH96 (IT = 3/S), 8: CH155 (IT = 0;/R), 9: CH157 (IT = 3/S), 10: CH159 (IT = 0;/R), 11: CH161 (IT = 0/R). Seedling IT values of 0-2 indicate resistance and 3-4 susceptibility. **(b)** Responses to a mixture of powdery mildew races under natural field conditions at the heading stage. The results of heading stage resistance evaluation are as follows: 1: *Th. ponticum* (IT = 0/R), 2: SY225 (IT = 9/S), 3: 7182 (IT = 7/S), 4: Abb (IT = 7/S), 5: CH88 (IT = 5/S), 6: CH94 (IT = 6/S), 7: CH96 (IT = 7/S), 8: CH155 (IT = 0;/R), 9: CH157 (IT = 6/S), 10: CH159 (IT = 6/S), 11: CH161 (IT = 0/R). Heading IT values of 0-4 indicate resistance and 5-9 susceptibility. R and S refer to resistance and susceptibility, respectively.

Resistance to powdery mildew was evaluated at both the seedling and heading stages over the 2023-2024 growing season. At the seedling stage, resistance was assessed using the *Bgt* isolate E09. Compare to susceptible control SY225, common wheat parent 7182 and Abb were fully infected, lines CH94 (2St), CH155 (4St), CH159 (6St) and CH161 (7St) showed resistance, while other derivatives were susceptible (**Figure 4a**). Resistance at the heading stage was evaluated in the field under natural infection conditions without artificial inoculation. CH155 and CH161, along with *Th. ponticum*, showed strong resistance, while the remaining lines and controls (SY225, 7182 and Abb) were susceptible (**Figure 4b**). The detailed data for the 2021-2022 and 2022-2023 growing seasons were provided in the supplementary figures (**Supplementary Figures S2**, **S3**); **Supplementary Table S2** and further supporting these observations.

### 3.4 Assessment of agronomic performance in seven derived lines

**Figure 5:**
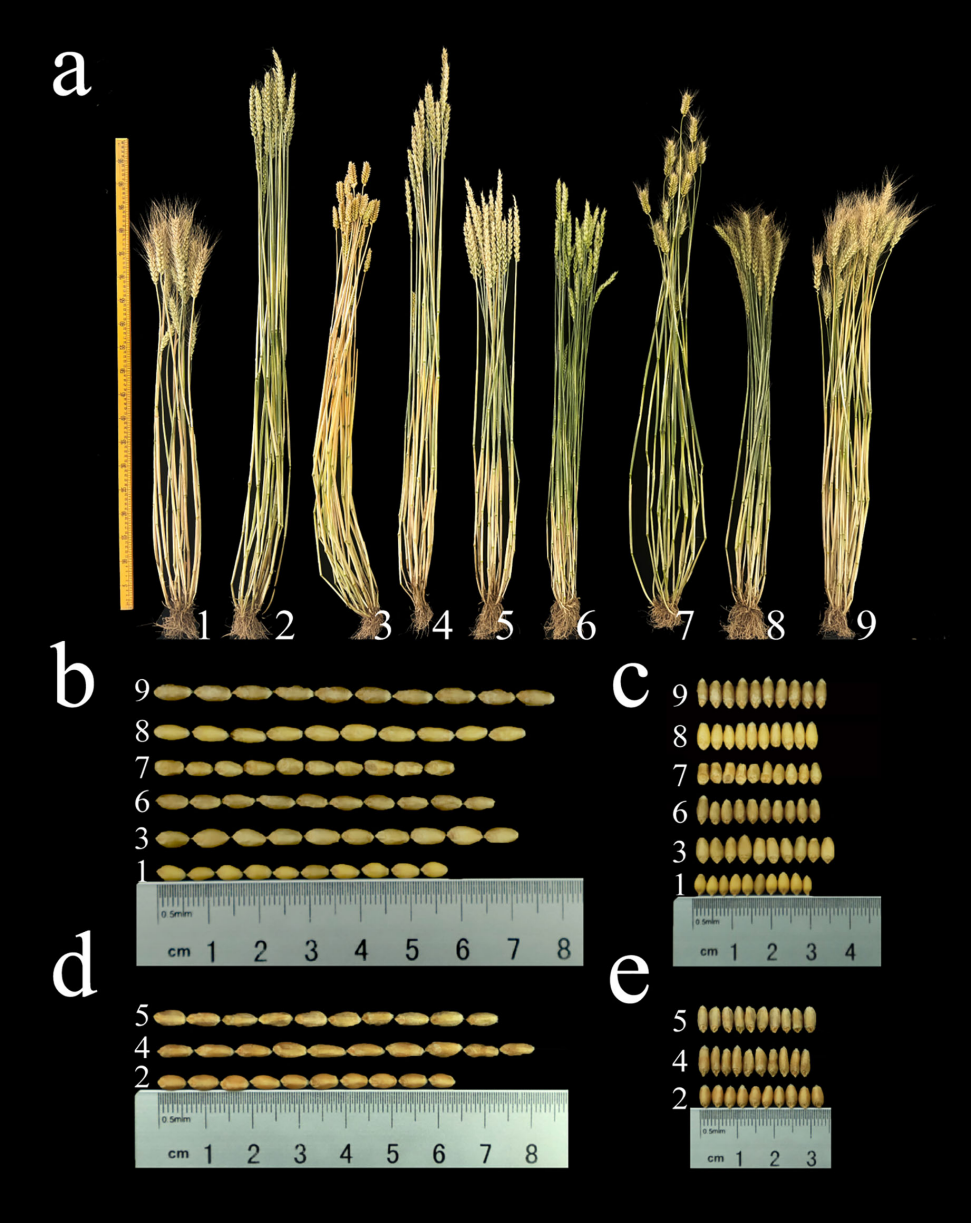
**(a)** Phenotypic images of the seven alien derivatives and their common wheat parental lines, along with 10-seed length **(b, d)** and width **(c, e)** images. 1: 7182, 2: Abb, 3: 1St (CH88), 4: 2St (CH94), 5: 3St (CH96), 6: 4St (CH155), 7: 5St (CH157), 8: 6St (CH159), 9: 7St (CH161).

From 2021 to 2024, we assessed the agronomic traits of seven derivative lines and their parental lines (**Supplementary Table S3**; **Figure 5**). Compared to the parent (7182/Abb), plant height of 1St and 5St significantly increased by 9.67 cm, 9.34 cm and 8.66 cm; 19.33 cm, 17.67 cm and 17.66 cm (p < 0.01) across the three growing seasons, respectively. In contrast, plant height in 3St and 7St significantly decreased by 15.33 cm, 15.33 cm and 15.00 cm; 10.67 cm, 11.00 cm and 11.34 cm (p < 0.01), respectively. Awn type assessment over three consecutive years showed that 7182, 5St, 6St and 7St exhibited long awns, while Abb, 2St, 3St and 4St displayed top awn and 1St exhibited awnless. In the spikelet number per spike analysis, only 3St showed a significant increase of 1.34 spikelets (p < 0.05) compared to Abb during the 2022-2023 growing season. No significant differences in spikelet number were observed for each derivative line compared to the parent. Analysis of thousand kernel weight revealed that 1St, 5St and 7St exhibited significant increases of 2.03 g, 2.55 g and 1.73 g; 2.92 g, 4.17 g and 4.19 g; 5.19 g, 5.19 g and 6.03 g, respectively, across all three growing seasons. In contrast, 2St, 3St, 4St and 6St exhibited reductions of 7.2 g, 6.84 g and 7.18 g; 15.26 g, 16.61 g and 16.08 g; 9.9 g, 8.02 g and 8.75 g; and 5.49 g, 3.42 g and 4.55 g respectively. Notably, 7St (CH161) exceeded a thousand-kernel weight of 41 g. In kernel length analysis, compared to parent line, only 7St showed significant increases across all three growing seasons, with increases of 0.129 cm, 0.101 cm and 0.103 cm, respectively; 1St and 6St showed significant increases of 0.049 cm and 0.043 cm, respectively, during the 2021-2022 season; in contrast, 3St showed a significant decrease in kernel length of 0.071 cm during the 2023-2024 season. In the kernel width analysis, only 1St and 3St showed significant increases across all three growing seasons, with increases of 0.048 cm, 0.043 cm and 0.032 cm for 1St, and 0.049 cm, 0.035 cm and 0.041 cm for 3St; 2St showed a significant increase of 0.02 cm only during the 2022-2023 season, while 6St showed a significant decrease of 0.028 cm only in the 2023-2024 season.

### 3.5 Development of *P. spicata* chromosome specific DNA markers

**Figure 6:**
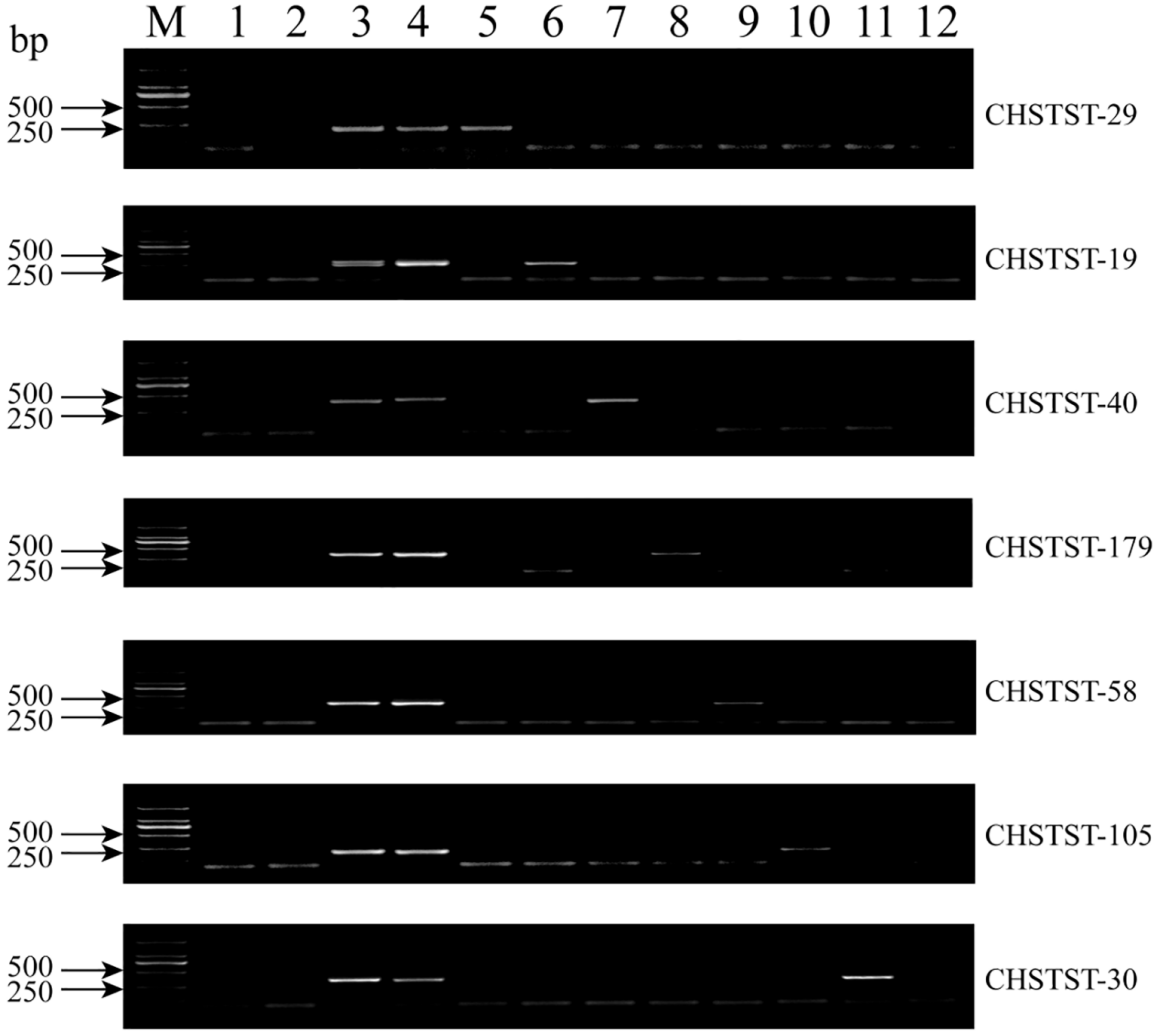
PCR amplification results of alien chromosome specific markers in 7182, Abb, *Th. ponticum*, *P.* sp*icata* and 1St -7St disomic derivatives. 1: 7182, 2: Abb, 3: *Th. ponticum*, 4: *P.* sp*icata*, 5: CH88, 6: CH94, 7: CH96, 8: CH155, 9: CH157, 10: CH159, 11: CH161, 12: H2O (negative control).

From the PE150 sequencing of *P.* sp*icata* (accession number CRA010636) using the MGISEQ platform (Deng et al., 2024), 3,075 candidate St chromosome sequences were identified. Of these, 179 sequences were selected for primer design and validation, yielding 85 primers that specifically amplified fragments from *Th. ponticum* and *P.* sp*icata*, while distinguishing them from 7182 and Abb (development efficiency: 47.5%). PCR amplification with gDNA from 7182, Abb, *Th. ponticum*, *P.* sp*icata* and the seven derivatives confirmed markers specific to each homoeologous group. For example, CHSTST-29 amplified bands similar to *Th. ponticum* and *P.* sp*icata* exclusively in CH88, indicating its specificity to the 1St chromosome of *P.* sp*icata* (**Figure 6**). In total, 29 markers were validated as specific to each St homoeologous group through agarose gel electrophoresis (**Supplementary Table S4**). The number of markers per line varied: CH96 (3St) had the highest at seven, while CH159 (6St) had the fewest at 2. CH88 (1St), CH157 (5St), and CH161 (7St) each had four markers, while CH94 (2St) and CH155 (4St) had 3 and 5 markers, respectively (**Table 1**).

**Table 1:** Specific markers for the complete set of St-group chromosome in *Th. ponticum*.

Group	Marker	7182	Abb	*Th. ponticum*	St	CH88	CH94	CH96	CH155	CH157	CH159	CH161	H_2_O
1	CHSTST-29	−	−	+	+	+	−	−	−	−	−	−	−
1	CHSTST-98	−	−	+	+	+	−	−	−	−	−	−	−
1	CHSTST-129	−	−	+	+	+	−	−	−	−	−	−	−
1	CHSTST-174	−	−	+	+	+	−	−	−	−	−	−	−
2	CHSTST-19	−	−	+	+	−	+	−	−	−	−	−	−
2	CHSTST-107	−	−	+	+	−	+	−	−	−	−	−	−
2	CHSTST-109	−	−	+	+	−	+	−	−	−	−	−	−
3	CHSTST-38	−	−	+	+	−	−	+	−	−	−	−	−
3	CHSTST-40	−	−	+	+	−	−	+	−	−	−	−	−
3	CHSTST-42	−	−	+	+	−	−	+	−	−	−	−	−
3	CHSTST-96	−	−	+	+	−	−	+	−	−	−	−	−
3	CHSTST-97	−	−	+	+	−	−	+	−	−	−	−	−
3	CHSTST-110	−	−	+	+	−	−	+	−	−	−	−	−
3	CHSTST-161	−	−	+	+	−	−	+	−	−	−	−	−
4	CHSTST-93	−	−	+	+	−	−	−	+	−	−	−	−
4	CHSTST-166	−	−	+	+	−	−	−	+	−	−	−	−
4	CHSTST-170	−	−	+	+	−	−	−	+	−	−	−	−
4	CHSTST-177	−	−	+	+	−	−	−	+	−	−	−	−
4	CHSTST-179	−	−	+	+	−	−	−	+	−	−	−	−
5	CHSTST-58	−	−	+	+	−	−	−	−	+	−	−	−
5	CHSTST-61	−	−	+	+	−	−	−	−	+	−	−	−
5	CHSTST-99	−	−	+	+	−	−	−	−	+	−	−	−
5	CHSTST-141	−	−	+	+	−	−	−	−	+	−	−	−
6	CHSTST-105	−	−	+	+	−	−	−	−	−	+	−	−
6	CHSTST-139	−	−	+	+	−	−	−	−	−	+	−	−
7	CHSTST-30	−	−	+	+	−	−	−	−	−	−	+	−
7	CHSTST-82	−	−	+	+	−	−	−	−	−	−	+	−
7	CHSTST-137	−	−	+	+	−	−	−	−	−	−	+	−
7	CHSTST-144	−	−	+	+	−	−	−	−	−	−	+	−

The symbols ‘+’and ‘−’ indicate the presence and absence of specific marker loci, respectively.

## 4 Discussion

As a self-pollinating crop, wheat evolves too slowly to meet the demands of modern agriculture. Introducing genetic material from related species is essential for breeding improved varieties (Wang et al., 2020b; Jiang et al., 1994; Zhang et al., 2022). *Th. ponticum*, a close relative of wheat, has been an important donor in wheat improvement for decades (Li and Wang, 2009), contributing genes that enhance disease resistance, yield, and other agronomic traits (Liu et al., 2018; Yang et al., 2023). Powdery mildew is a major fungal disease threatening wheat production, with the effectiveness of existing resistance genes diminishing over time (Huang et al., 2023; Wu et al., 2023; Lu et al., 2024). To date, *Pm51* is the only officially named powdery mildew resistance gene derived from *Th. ponticum* in wheat (Zhan et al., 2014). Thus, *Th. ponticum* still holds untapped genetic resources for powdery mildew resistance.

We evaluated seven derived lines in this study. Seedling resistance was assessed using the E09 isolate through artificial inoculation, while field trials relied on natural infections. The 4St and 7St derivatives exhibited high resistance at both stages, showing resistance to the E09 at the seedling stage and to the natural pathogen isolates in the field at the heading stage. However, the 4St line showed a significant decrease in thousand-kernel weight compared to the parent line 7182, while no significant difference in plant height was observed. In contrast, the 7St line showed a decrease in plant height, while significantly increasing both thousand-kernel weight and kernel length. The 1St, 3St and 5St derivatives were susceptible to powdery mildew at both stages. Notably, while thousand-grain weight and plant height in the 1St and 5St derivatives were significantly higher than in the parent, the height increase is undesirable for wheat breeding. This suggests that the introduction of alien chromosomal fragments may improve certain traits while potentially introducing unfavorable genes (Han et al., 2024a; Veyrieras et al., 2007). The 2St and 6St lines were resistant at the seedling stage but susceptible at the heading stage. This stage-specific resistance is likely attributable to differences in pathogen isolates, with the E09 isolate used at the seedling stage differing from the naturally occurring mixed pathogen populations at the heading stage. To analyze resistance differences between seedling and adult stages and eliminate pathogen and environmental variations, future studies will use the same pathogen isolate for resistance evaluation at both stages. Additionally, the 2St and 6St derivatives showed a significant reduction in thousand-kernels weight. Overall, the 7St derivative line represents an ideal case of introgression, exhibiting high resistance to powdery mildew. Plant height was reduced to 71.3 cm, while thousand-kernel weight and kernel length increased to 41 g and 0.77 cm, respectively, highlighting its value as breeding material. Although the introduction of alien genes can enhance disease resistance, it also carries the risk of undesirable traits. Therefore, molecular marker-assisted selection (MAS), combined with backcrossing and genomic selection (GS), effectively reduces the fixation of unfavorable genes while balancing disease resistance and yield traits. Moreover, radiation-induced small fragment translocations provide a novel method for rapid gene locus identification and the accumulation of target traits. When combined with CRISPR/Cas9 gene editing, this approach could expedite the breeding process.

Accurate identification and evaluation of wheat-alien chromosomes are critical for efficiently utilizing germplasm materials (Liu et al., 2022; Bian et al., 2018). FISH and GISH techniques enable the precise identification of chromosomal structural variations and interspecies hybridization events (Han et al., 2024b; An et al., 2022). Previous studies revealed variability in FISH patterns of the St subgenome in *Th. ponticum* and *Th. intermedium* (Linc et al., 2017; Qi et al., 2023). In this study, FISH analysis of CH157 (5St) showed the loss of Oligo-pSc119.2-1 signal at the distal end of chromosome 6BL, along with the unexpected presence of Oligo-pSc119.2-1 signal at the distal end of chromosome 6DL, suggesting a possible reciprocal translocation between 6BL and 6DL. Additionally, a strong Oligo-pSc119.2-1 signal was detected at the distal end of chromosome 6AS, while chromosome 4BS exhibited breakage and loss of the signal, suggesting a translocation of the 4BS fragment to 6AS (**Figure 1e2**). Similar rearrangements have been detected in other wheat derivatives using advanced techniques such as ND-FISH and oligo-FISH painting (Li et al., 2021; Wang et al., 2023). These chromosomal rearrangements are common in the Triticeae tribe, with dynamic changes frequently occurring in telomeric and centromeric regions (Oizumi et al., 2021). Through analysis of the addition lines of Xiaoyan 7430 (a wheat-*Th. ponticum* partial amphiploid), it was found to carry alien chromosomes 1St, 3St, 4E, 5St, 6St and 7St. Comparison of the St karyotypes of these alien chromosomes with the corresponding St homoeologous group karyotypes in this study showed consistent signal patterns. However, the 4E chromosome in Xiao Yan 7430, compared to the 4St karyotype in this study, was missing the 119.2 signal at the terminal end of the short arm (Jia et al., 2022). This suggests that although some homoeologous groups are identical, chromosome-set differences can lead to variations in the karyotype.

The narrow genetic base of wheat necessitates hybridization with related species to introduce desirable traits (Li et al., 2023). Traditional cytogenetic methods for identifying exogenous chromosomes are time-consuming and labor-intensive. Marker-assisted selection offers a faster alternative. Using SLAF-seq, Han developed 404 universal and KASP markers for rye chromosome arms, enabling efficient detection of rye chromatin in wheat (Han et al., 2020). In this study, specific markers for 1-7St chromosomes of *Th. ponticum* were developed, facilitating precise tracking of St chromosomes in a wheat background. The markers for 4St and 7St are particularly valuable for breeding powdery mildew-resistant varieties, providing molecular tools for efficient disease resistance breeding.

## 5 Conclusion

This study employed cytogenetic and liquid array analyses to establish a comprehensive karyotype of the St-group chromosomes in seven derivatives. Resistance to powdery mildew was assessed using the E09 isolate at the seedling stage and natural field infections at the heading stage. Resistance to powdery mildew was assessed with the E09 isolate at the seedling stage and natural field infections at the heading stage, with 4St and 7St showing high resistance at both growth stages. Agronomic trait evaluation revealed that the 7St derivative line exhibited a plant height reduction to 71.3 cm, while thousand-kernel weight and kernel length were increased to 41 g and 0.77 cm, respectively. Furthermore, specific markers for each St-homoeologous group were developed with an efficiency of 47.5%. These seven derivative lines lay the foundation for research on the *Th. ponticum* St subgenome. Through crosses with conventional wheat varieties and subsequent phenotypic and molecular selection of their progeny, they hold promise for integration into wheat breeding programs.

## Data Availability

The sequencing data in this manuscript were sourced from the National Genomics Data Center (NGDC) of the China National Center for Bioinformation (CNCB) database (accession number: CRA010636).

## Supplementary material

Supplementary Figure 1 mc-GISH with *P.* sp*icata* gDNA (green) and *Th. elongatum* gDNA (red) as probes. c3-g3 are mc-GISH patterns of derivatives. (c3: CH96, d3:CH155, e3: CH157, f3: CH159, g3: CH161, Bar = 10 μm.) Arrows denote alien chromosomes.

Supplementary Figure 2 Evaluation of powdery mildew resistance in seven wheat-*Th. ponticum* alien derivatives and their parental lines in 2021-2022. **(a)** Responses to the *Bgt* isolate E09 at the seedling stage. The results of seedling stage resistance evaluation are as follows: 1: *Th. ponticum* (IT = 0/R), 2: SY225 (IT = 4/S), 3: 7182 (IT = 3/S), 4: Abb (IT = 3/S), 5: CH88 (IT = 3/S), 6: CH94 (IT = 0/R), 7: CH96 (IT = 3/S), 8: CH155 (IT = 0;/R), 9: CH157 (IT = 3/S), 10: CH159 (IT = 0;/R), 11: CH161 (IT = 0/R). Seedling IT values of 0-2 indicate resistance and 3-4 susceptibility. **(b)** Responses to a mixture of powdery mildew races under natural field conditions at the heading stage. The results of heading stage resistance evaluation are as follows: 1: Th. ponticum (IT = 0/R), 2: SY225 (IT = 9/S), 3: 7182 (IT = 7/S), 4: Abb (IT = 6/S), 5: CH88 (IT = 6/S), 6: CH94 (IT = 5/S), 7: CH96 (IT = 7/S), 8: CH155 (IT = 0;/R), 9: CH157 (IT = 5/S), 10: CH159 (IT = 6/S), 11: CH161 (IT = 0/R). Heading IT values of 0-4 indicate resistance and 5-9 susceptibility. R and S refer to resistance and susceptibility, respectively.

Supplementary Figure 3 Evaluation of powdery mildew resistance in seven wheat-*Th. ponticum* alien derivatives and their parental lines in 2022-2023. **(a)** Responses to the *Bgt* isolate E09 at the seedling stage. The results of seedling stage resistance evaluation are as follows: 1: *Th. ponticum* (IT = 0/R), 2: SY225 (IT = 4/S), 3: 7182 (IT = 3/S), 4: Abb (IT = 3/S), 5: CH88 (IT = 3/S), 6: CH94 (IT = 0/R), 7: CH96 (IT = 3/S), 8: CH155 (IT = 0;/R), 9: CH157 (IT = 3/S), 10: CH159 (IT = 0;/R), 11: CH161 (IT = 0/R). Seedling IT values of 0-2 indicate resistance and 3-4 susceptibility. **(b)** Responses to a mixture of powdery mildew races under natural field conditions at the heading stage. The results of heading stage resistance evaluation are as follows: 1: *Th. ponticum* (IT = 0/R), 2: SY225 (IT = 8/S), 3: 7182 (IT = 6/S), 4: Abb (IT = 6/S),5: CH88 (IT = 5/S), 6: CH94 (IT = 6/S), 7: CH96 (IT = 7/S), 8: CH155 (IT = 0/R), 9: CH157 (IT = 6/S), 10: CH159 (IT = 7/S), 11: CH161 (IT = 0/R). Heading IT values of 0-4 indicate resistance and 5-9 susceptibility. R and S refer to resistance and susceptibility, respectively.
