# Supplementary figures and images for: Establishment of a set of St-group wheat-Thinopyrum ponticum derivative lines conferring resistance to powdery mildew
Source: Front Plant Sci. 2025 Apr 16;16:1576050. doi: 10.3389/fpls.2025.1576050 (PMC12044885; doi:10.3389/fpls.2025.1576050)

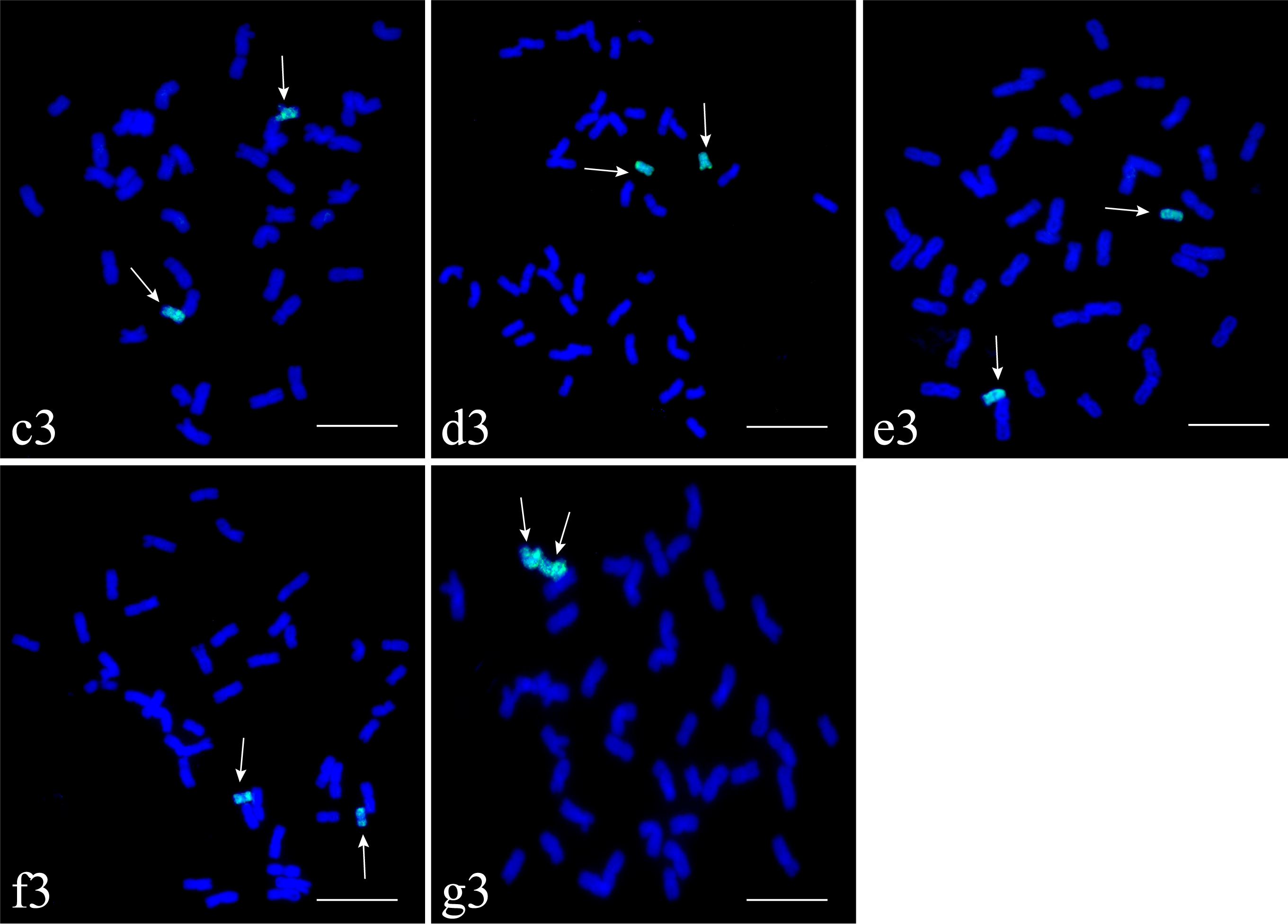

Supplement: Supplementary Figure 1 — mc-GISH with P. spicata gDNA (green) and Th. elongatum gDNA (red) as probes. c3-g3 are mc-GISH patterns of derivatives. (c3: CH96, d3:CH155, e3: CH157, f3: CH159, g3: CH161, Bar = 10 μm.) Arrows denote alien chromosomes. [file Image1.png]

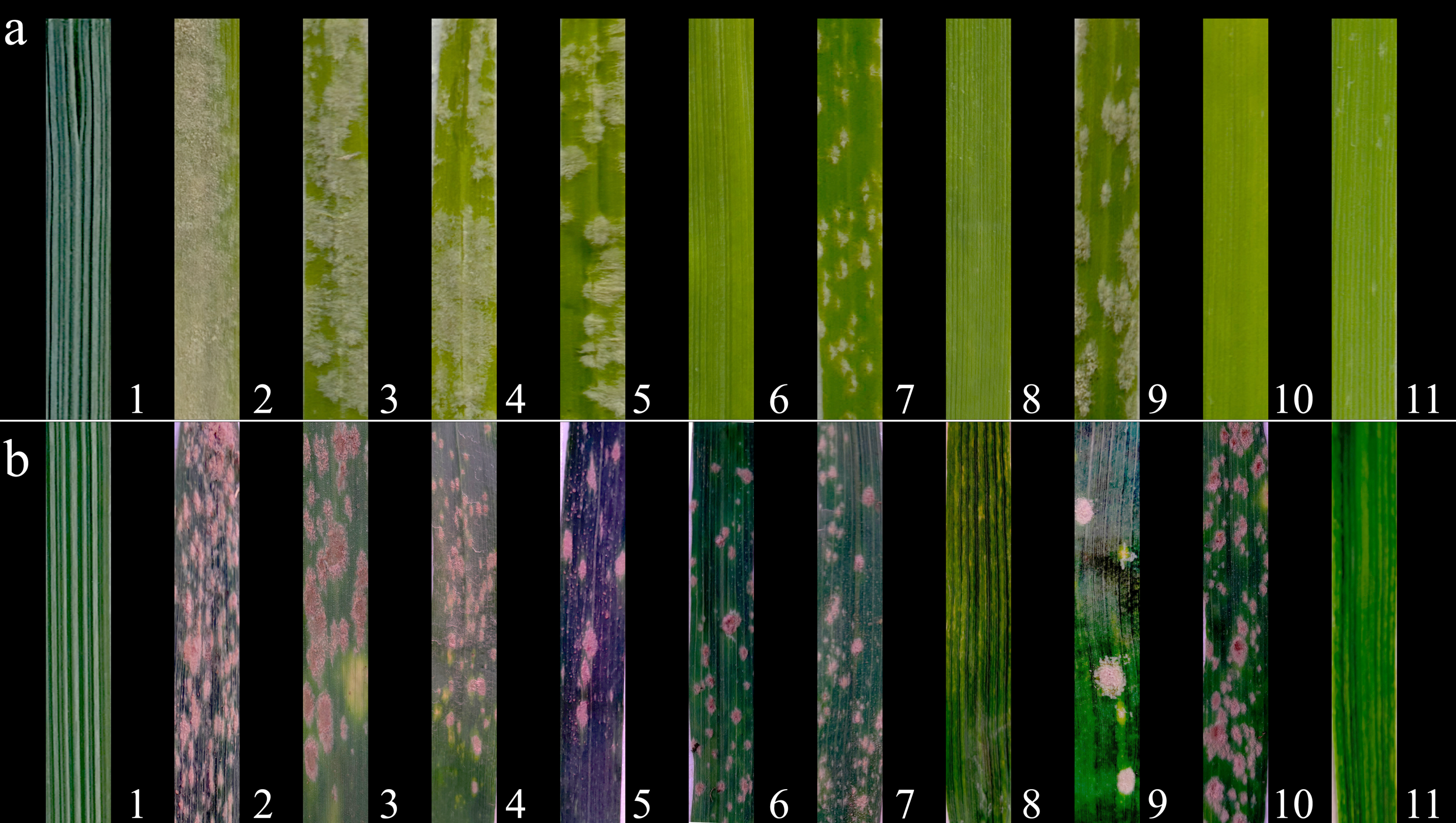

Supplement: Supplementary Figure 2 — Evaluation of powdery mildew resistance in seven wheat-Th. ponticum alien derivatives and their parental lines in 2021-2022. (a) Responses to the Bgt isolate E09 at the seedling stage. The results of seedling stage resistance evaluation are as follows: 1: Th. ponticum (IT = 0/R), 2: SY225 (IT = 4/S), 3: 7182 (IT = 3/S), 4: Abb (IT = 3/S), 5: CH88 (IT = 3/S), 6: CH94 (IT = 0/R), 7: CH96 (IT = 3/S), 8: CH155 (IT = 0;/R), 9: CH157 (IT = 3/S), 10: CH159 (IT = 0;/R), 11: CH161 (IT = 0/R). Seedling IT values of 0-2 indicate resistance and 3-4 susceptibility. (b) Responses to a mixture of powdery mildew races under natural field conditions at the heading stage. The results of heading stage resistance evaluation are as follows: 1: Th. ponticum (IT = 0/R), 2: SY225 (IT = 9/S), 3: 7182 (IT = 7/S), 4: Abb (IT = 6/S), 5: CH88 (IT = 6/S), 6: CH94 (IT = 5/S), 7: CH96 (IT = 7/S), 8: CH155 (IT = 0;/R), 9: CH157 (IT = 5/S), 10: CH159 (IT = 6/S), 11: CH161 (IT = 0/R). Heading IT values of 0-4 indicate resistance and 5-9 susceptibility. R and S refer to resistance and susceptibility, respectively. [file Image2.png]

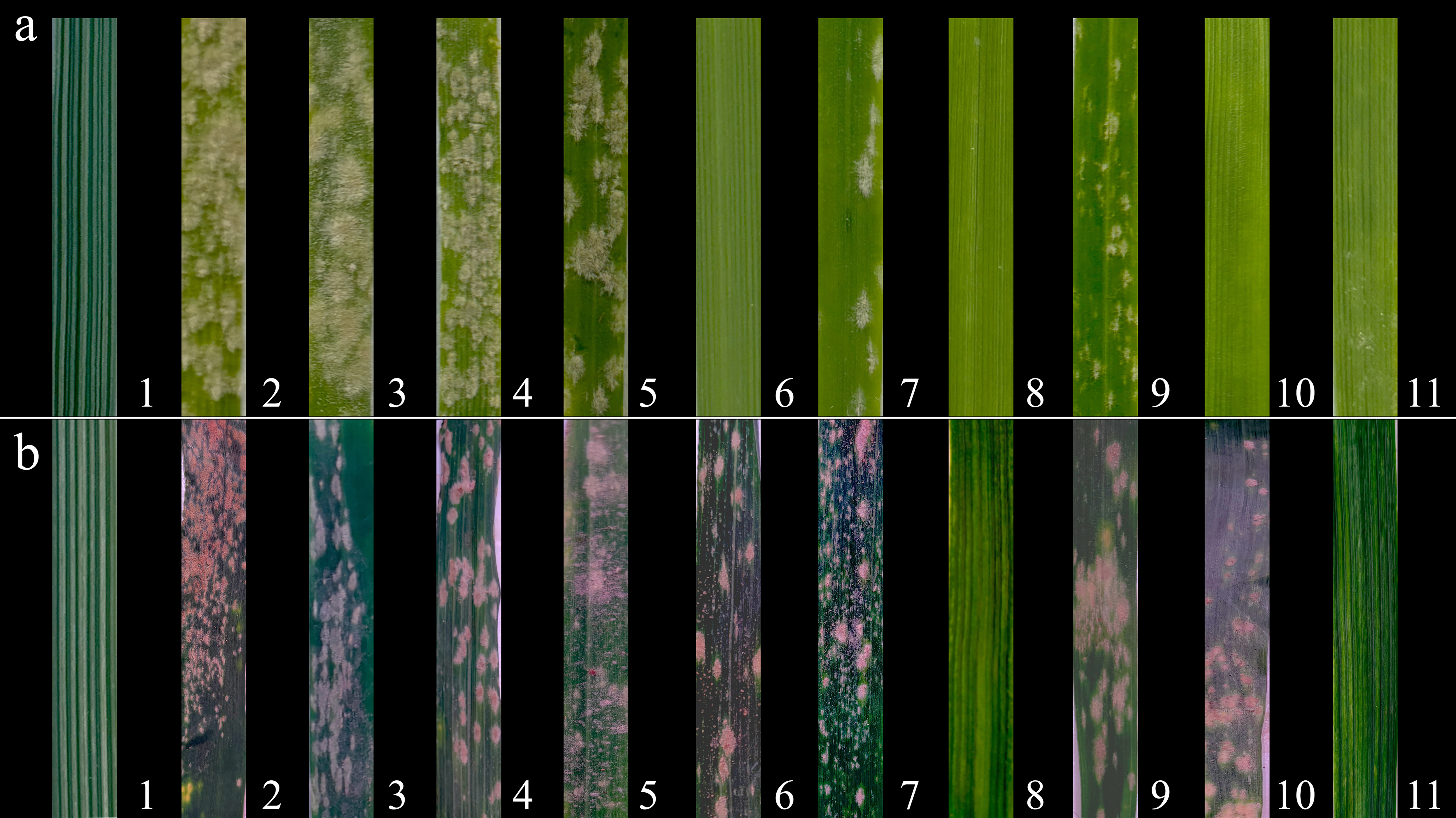

Supplement: Supplementary Figure 3 — Evaluation of powdery mildew resistance in seven wheat-Th. ponticum alien derivatives and their parental lines in 2022-2023. (a) Responses to the Bgt isolate E09 at the seedling stage. The results of seedling stage resistance evaluation are as follows: 1: Th. ponticum (IT = 0/R), 2: SY225 (IT = 4/S), 3: 7182 (IT = 3/S), 4: Abb (IT = 3/S), 5: CH88 (IT = 3/S), 6: CH94 (IT = 0/R), 7: CH96 (IT = 3/S), 8: CH155 (IT = 0;/R), 9: CH157 (IT = 3/S), 10: CH159 (IT = 0;/R), 11: CH161 (IT = 0/R). Seedling IT values of 0-2 indicate resistance and 3-4 susceptibility. (b) Responses to a mixture of powdery mildew races under natural field conditions at the heading stage. The results of heading stage resistance evaluation are as follows: 1: Th. ponticum (IT = 0/R), 2: SY225 (IT = 8/S), 3: 7182 (IT = 6/S), 4: Abb (IT = 6/S),5: CH88 (IT = 5/S), 6: CH94 (IT = 6/S), 7: CH96 (IT = 7/S), 8: CH155 (IT = 0/R), 9: CH157 (IT = 6/S), 10: CH159 (IT = 7/S), 11: CH161 (IT = 0/R). Heading IT values of 0-4 indicate resistance and 5-9 susceptibility. R and S refer to resistance and susceptibility, respectively. [file Image3.jpeg]
